# Impact of oncogenic viruses on autoimmune diseases and tumorigenesis

**DOI:** 10.1186/s13027-026-00746-7

**Published:** 2026-03-18

**Authors:** Vahideh Hamidi Sofiani, Fatemeh Khodadadpour Mahani, Niloofar Farsiu, Maysam Yousefi, Malihe Naderi, Ghazal Ahmadi, Mohsen Nakhaie

**Affiliations:** 1 Department of Microbiology, Faculty of Medicine, Golestan University of Medical Science, Gorgan, Iran; 2 Research Center of Tropical and Infectious Diseases, Kerman University of Medical Sciences, Kerman, Iran; 3 Gastroenterology and Hepatology Research Center, Institute of Basic and Clinical Physiology Sciences, Kerman University of Medical Sciences, Kerman, Iran; 4 Social Determinants of Health Research Center, Institute for Futures Studies in Health, Kerman University of Medical Sciences, Kerman, Iran; 5 Department of Tropical Viral Vaccine Development, Institute of Tropical Medicine, Nagasaki University, Nagasaki, Japan; 6 Department of Microbiology and Microbial Biotechnology, Faculty of Life Sciences and Biotechnology, Shahid Beheshti University, Tehran, Iran; 7 Department of Microbiology, School of Medicine, Kerman University of Medical Sciences, Kerman, Iran; 8 Clinical Research Development Unit, Afzalipour Hospital, Kerman University of Medical Sciences, Kerman, Iran

**Keywords:** Oncogene Virus, Autoimmune Disease, Cancer

## Abstract

Autoimmune diseases occur when the immune system loses its ability to differentiate self from non-self, resulting in a breakdown of immune tolerance. Their development is shaped by a combination of genetic predisposition and environmental triggers, among which viral infections play a key role. Oncogenic viruses are of particular interest due to their dual involvement in both cancer development and autoimmune responses. The overlapping molecular mechanisms between autoimmunity and cancer suggest a complex interplay that requires further exploration. Interestingly, autoimmune diseases and early-stage cancers often share inflammatory features, whereas advanced cancers tend to exhibit an opposing immune environment. This contrast may help explain the higher incidence of cancer and the lower cancer-specific mortality observed in patients with pre-existing autoimmune disorders. This review explores the current understanding of oncogenic viruses and their role in linking autoimmune diseases and cancer, emphasizing the importance of shared molecular pathways between these conditions.

## Introduction

Autoimmune diseases occur when the immune system falsely recognizes the host cells’ antigens as foreign or a threat, leading to immune response stimulation and attacking normal functioning host cells. Currently, more than 80 distinct autoimmune diseases have been identified [1]. Given the complex nature of autoimmune disease pathogenesis, developing effective therapies remains a challenge [2]. Therefore, a deeper understanding of the involved cell types and mediators is essential for identifying potential targets for future treatments. While the exact causes for many of these conditions remain unclear, various factors such as genetics, age, and environmental factors have been linked to the initiation and progression of autoimmune diseases [3]. Among environmental factors, viruses are particularly significant, as they often initiate autoimmune reactions, particularly in genetically predisposed individuals [4, 5]. One notable example is the severe acute respiratory syndrome coronavirus 2 (SARS-CoV-2), the virus responsible for the COVID-19 pandemic, which has affected millions of people worldwide [6]. Multiple reports have indicated the onset of autoimmune diseases following SARS-CoV-2 infection [7]. These findings highlight the potential role of viral infections as triggers in the development of autoimmune diseases. A deeper understanding of the mechanisms through which viruses can initiate autoimmune-related dysregulations can aid in the battle against these conditions.

A primary strategy explored and applied in managing autoimmune conditions in humans is enhancing immune tolerance. Immune tolerance refers to the immune system’s state of non-reactivity toward self-tissues, while maintaining the ability to recognize and attack foreign and harmful antigens. Several mechanisms contribute to the establishment and maintenance of this state, including the elimination of self-reactive receptors in the bone marrow and thymus (central tolerance). However, not all self-reactive cells are removed in the primary lymphoid organs. For example, the pool of naïve T cells exiting the thymus contains a substantial proportion of low-avidity self-reactive cells, which may potentially initiate autoimmune responses. Thus, additional mechanisms of peripheral tolerance are induced to prevent their activation [8]. Specialized cell subsets such as regulatory T cells (Tregs), regulatory B cells (Bregs), tolerogenic dendritic cells (tolDCs), and M2 macrophages play vital roles in maintaining the balance between tolerance and immune activation. However, this delicate equilibrium can be disrupted by genetic predisposition, epigenetic modifications, and environmental factors, leading to autoimmune diseases [9]. Consequently, increasing attention has been directed toward strategies that enhance immune tolerance.

Although immune tolerance is critically important to avoid autoimmunity, it can also be exploited by cancerous cells to create an immunosuppressive environment. The recruitment of tolerogenic cell subsets and the evasion of immune responses are hallmark features of malignancies. Cancer cells may express immune checkpoint proteins, impair antigen presentation, undergo epithelial-to-mesenchymal transition (EMT), or exhibit changes in RNA editing. As a result, tumor-specific antigens (TSAs) or tumor-associated antigens (TAAs) often fail to provoke an effective immune response [10]. Therefore, considerable efforts have been dedicated to overcoming cancer-induced tolerance and mobilizing the immune system to combat cancer [11, 12].

Remarkably, some viruses make proteins that can directly disrupt the cell cycle control, DNA repair, and apoptosis. Some encourage long-term inflammation, immune evasion, and persistent infection, which create a microenvironment conducive to cancer development. Oncogenic viruses (oncoviruses) are viruses that cause cancer by changing the pathways of host cells directly or indirectly [13, 14]. Oncoviruses are also associated with autoimmune diseases. As a result, they can be a unique connection between cancer and autoimmunity. These viruses can cause malignant transformation and disturb self-tolerance simultaneously by encouraging immune evasion and chronic inflammation. This increases the likelihood of autoimmune manifestations in people [15]. Viral proteins might disrupt antigen presentation, modify cytokine profiles, or initiate molecular mimicry, resulting in a dual effect: inhibiting effective anti-tumor immunity while provoking inappropriate immune responses against host tissues [16]. This duality highlights the necessity of investigating oncogenic viruses not only in relation to cancer but also as potential factors in autoimmune pathogenesis. Comprehending the mechanisms by which viruses undermine tolerance and alter immune equilibrium may facilitate the development of targeted therapies that prevent both autoimmunity and tumor progression. This review examines the interrelated roles of significant oncoviruses in the pathogenesis of autoimmune diseases and cancer, emphasizing the mechanisms by which viral infections undermine immune tolerance, induce autoimmunity, and promote tumor progression.

## Human papillomavirus (HPV)

HPV is one of the most prevalent sexually transmitted infections worldwide [17]. With over 200 identified types, HPV is categorized based on tissue tropism and its potential to transform epithelial cells [18]. The majority of HPV infections are either asymptomatic or associated with mild symptoms and often resolve spontaneously within two years [19]. However, in approximately 5% to 10% of infected women, HPV integrates into the host genome. This integration typically involves the deletion of the E2 regulatory gene, leading to the overexpression of the viral oncogenes E6 and E7. These oncogenes target essential tumor suppressor proteins, p53 and Rb, disrupting critical cellular processes such as cell cycle regulation, cell adhesion, and apoptosis. The integration of viral DNA into the host genome provides a selective growth advantage to epithelial cells, thereby increasing the risk of persistent infection, development of cellular abnormalities, cervical lesions, and ultimately cervical cancer [20]. HPV affects several immune-modulatory and tumorigenic pathways through epigenetic modifications, including altered microRNA expression (e.g., miR-22, miR-20b), telomerase activation via hTERT induction by C-Myc, inhibition of TGF-β and NOTCH signaling, and centrosome duplication errors and chromosomal instability via E7 [21, 22]. These combined molecular effects promote immune evasion, carcinogenesis, and potentially autoimmunity in susceptible hosts.

### HPV role in tumorigenesis

HPV is one of the most prevalent oncogenic viruses, playing a well-established role in various cancers, including cervical, anal, colorectal, gastric, esophageal, bladder, and head and neck cancers. High-risk HPV genotypes, primarily types 16 and 18, are the main contributors to malignancy. HPV oncoproteins interfere with crucial tumor suppressors. Specifically, E6 promotes the proteasomal degradation of p53 by interacting with the E6-associated protein (E6AP) and disrupting p300/CBP co-activator complexes, while E7 binds to pRb, releasing E2F transcription factors and driving cell cycle progression. In addition to these actions, E6 and E7 interfere with other regulatory elements such as p21, p27, and Notch1 signaling, which contributes to genomic instability and oncogenesis. Furthermore, HPV promotes telomerase activation via hTERT and disrupts apoptosis by interfering with Fas-mediated signaling pathways. Beyond its oncogenic potential, HPV infection may also contribute to autoimmune dysregulation. Viral proteins such as E6 and E7 not only disrupt tumor suppressor pathways but can also alter immune signaling networks, including interferon responses, cytokine production, and antigen presentation. These changes promote chronic immune activation and may break self-tolerance in genetically predisposed individuals [23–25].

### HPV role in autoimmune diseases

While there have been multiple studies evaluating the effect of HPV infection on autoimmune diseases, many of these studies have remained epidemiological, and the exact mechanisms of viral infection leading to these conditions are still not precisely known. However, the association between HPV infection and autoimmune diseases has been reported in various studies. For instance, a study provided computational evidence that high-risk HPV peptides look like human lupus-related proteins and can bind strongly to immune system receptors, suggesting that HPV infection could trigger or worsen lupus via molecular mimicry [26]. Interestingly enough, studies have also shown a higher prevalence of high-risk HPV types, multiple HPV infections, and an increased incidence of high-grade cervical dysplasia in individuals with systemic lupus erythematosus (SLE) [27], suggesting that autoimmune conditions and their treatments can influence the risk of HPV infection, making women with autoimmune diseases more prone to cervical dysplasia [28]. However, no significant increases in cervical cancer risk have been observed in women with SLE in some studies [27, 29, 30].

The relationship between rheumatoid arthritis (RA) and HPV infection has also been explored. Some reports indicate a higher frequency of abnormal cervical cytology related to HPV in RA patients. However, there is no conclusive evidence linking persistent HPV infection, increased risk of high-grade dysplasia, or cervical cancer in these cases [31]. One proposed explanation links persistent HPV-18 infection delivering viral antigens that mimic joint-associated self-antigens to activating autoreactive B and T cells and promoting the production of anti-citrullinated protein antibodies, ultimately leading to synovial inflammation. A recent study conducted in the UK observed a positive nominal association between HPV-18 and both prevalent T1D and incident RA [32]. HPV infection can also aggravate rheumatoid arthritis (RA) by driving chronic systemic inflammation. Viral oncoproteins such as E5, E6, and E7 upregulate COX-2/PGE2 signaling and induce oxidative stress, which amplify pro-inflammatory cytokine networks and disrupt immune regulation. This sustained inflammatory state reinforces synovial inflammation in RA, potentially worsening disease manifestations and progression [33]. A cross-sectional study based on five cycles of 2-year surveys also reported a positive association between HPV infection and RA incident, and a lower incident of the disease associated with HPV vaccination [34], Showcasing the benefit of vaccination in protection against HPV-induced RA.

While HPV vaccination has saved many lives and protected many against cervical cancer related to HPV infection, it has also been shown that HPV vaccination can lead to autoimmune diseases in rare cases. More than a decade since the approval of the first HPV vaccines, mounting evidence supports the widespread adoption of HPV immunization programs. Clinical trials and post-marketing observational studies consistently demonstrate the efficacy, effectiveness, and safety of the accessible HPV vaccines [35, 36]. However, an analysis of 12 patients’ case reports of SLE development after HPV vaccination revealed that 7 cases of SLE developed between 3 weeks and 2 months post-HPV vaccination, suggesting that a personal or family history of autoimmune diseases should be considered before vaccination [37].

**Fig. 1:**
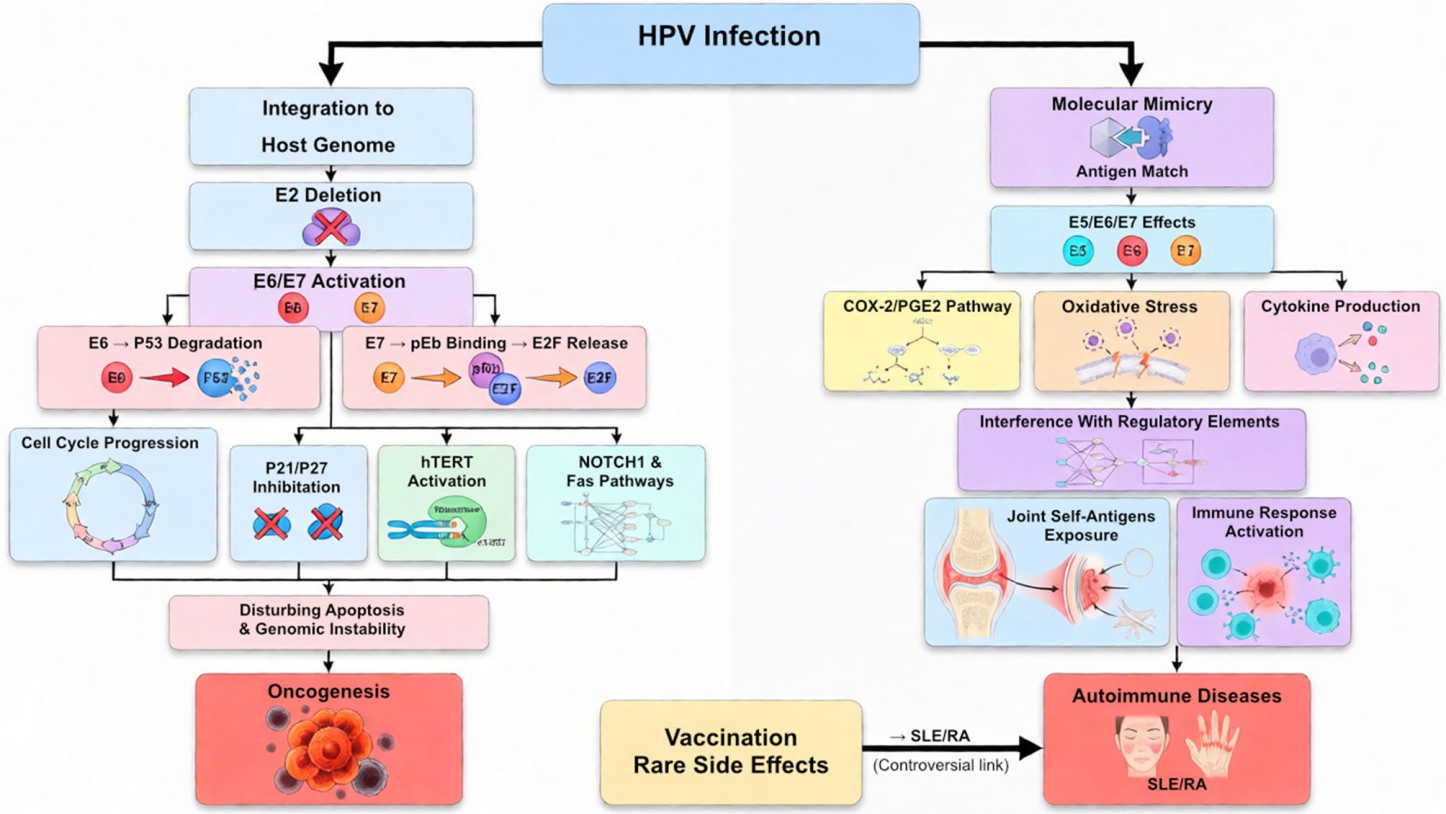
Key pathways of HPV infection contributing to cancer and autoimmunity and rare serious side effects of HPV vaccines have also been mentioned. SLE: systemic lupus erythematosus (SLE), RA: rheumatoid arthritis

Another autoimmune disease, known as Guillain-Barré syndrome (GBS), stands as one of the more severe potential risks associated with vaccination. GBS is an uncommon autoimmune disorder characterized by the immune system attacking nerve cells, leading to muscle weakness and, in severe cases, paralysis. Its occurrence is less than one case per 100,000 person-years among individuals aged 10–19 years, the relevant age group for HPV vaccination [38]. The precise causes of GBS remain unclear, but it frequently follows infections of viruses and bacteria and, in uncommon instances, vaccination [39]. Although various clinical studies did not establish a link between HPV vaccination and GBS incidence, a 2017 French study described a more than threefold elevated relative risk [40, 41] (Fig. 1).

## Epstein-barr virus (EBV)

EBV is a lymphotropic virus first identified in cells isolated from African Burkitt’s lymphoma, and later recognized as a globally prevalent virus [42, 43]. It is a widespread infectious agent, with nearly 95% of the global population harboring its latent infections [44]. Transmission primarily occurs through saliva, with initial replication at mucosal surfaces—particularly in the oropharyngeal and nasopharyngeal epithelial cells, especially in the tonsillar region. The virus then infiltrates underlying tissues and infects resting B cells by binding its viral envelope glycoprotein 350 (gp350) to the B-cell type 2 complement receptor (CD21) [45].

A key aspect of EBV’s disease biology is its ability to switch between an active lytic cycle and a latent state, with periodic reactivation from latency [46]. Chronic relapsing EBV infection and impaired immune control of the virus have been linked to several systemic autoimmune diseases (SADs) and multiple sclerosis (MS) [35]. For example, numerous studies have demonstrated a strong association between EBV infection and SLE. Nearly all adult SLE patients (99.5%) are infected with EBV [44]. However, this finding’s statistical significance is somewhat tempered by the high prevalence of EBV infection among healthy adults as well, which stands at approximately 95%. In individuals younger than 20 years old, the difference between SLE patients and healthy controls is more pronounced. A study showed that about 70% of healthy people were infected compared to nearly all young SLE patients (99.6%) [47].

SLE patients show a markedly higher number of EBV-infected peripheral B cells, with at least a tenfold increase compared to healthy individuals. This elevated presence correlates with increased disease activity in SLE and occurs independently of immunosuppressive treatments [48]. In Grave’s disease, it has been suggested that polyclonal B cell activation due to LMP1 enabled B cells to undergo class-switch recombination to produce every isotype of Ig, leading to autoantibody production, which contributes to the development and exacerbation of autoimmune diseases [49]. This may also be the case for SLE initiation in EBV-infected individuals. Additionally, several studies have found abnormally high EBV viral loads in peripheral blood mononuclear cells (PBMCs) of SLE patients compared to controls [50–52]. Moreover, a significantly higher level of EBV DNA has been detected in the serum of many SLE patients compared to healthy subjects [53]. Based on this information, it can be proposed that viral reactivation may contribute to the pathogenesis of SLE. Abnormal expression of EBV viral mRNAs such as EBNA, LMP-1, BZLF-1, and LMP-2 has been observed in PBMCs from SLE patients [54]. The levels of these mRNAs are often higher in SLE patients than in those with infectious mononucleosis (IM), indicating highly active EBV infection. Notably, BZLF-1, an early lytic gene essential for initiating viral replication, shows increased expression in SLE, supporting the idea of viral reactivation [55]. As for the part of latent EBV infection in SLE, similar to what was discussed earlier in the HPV section, a molecular mimicry model suggests that, due to the homology of EBNA1 to SLE, the anti-EBNA1 response leads to anti-EBNA1 antibodies cross-reacting with an SLE autoantigen though a molecular mimicry mechanism, causing autoantibody epitope spreading, and culminating in clinical SLE [56, 57].

EBV is also believed to have an important role in the development of RA. Although not all studies consistently show a direct connection between the two [58]. Molecular mimicry has also been reported between EBV and RA. The presence of anti-citrullinated protein antibodies (ACPAs) in a large number of RA patients has been confirmed. These antibodies specifically recognize a citrullinated region of EBNA2, an essential EBV transcription factor expressed during the virus’s lytic phase [59].

**Fig. 2:**
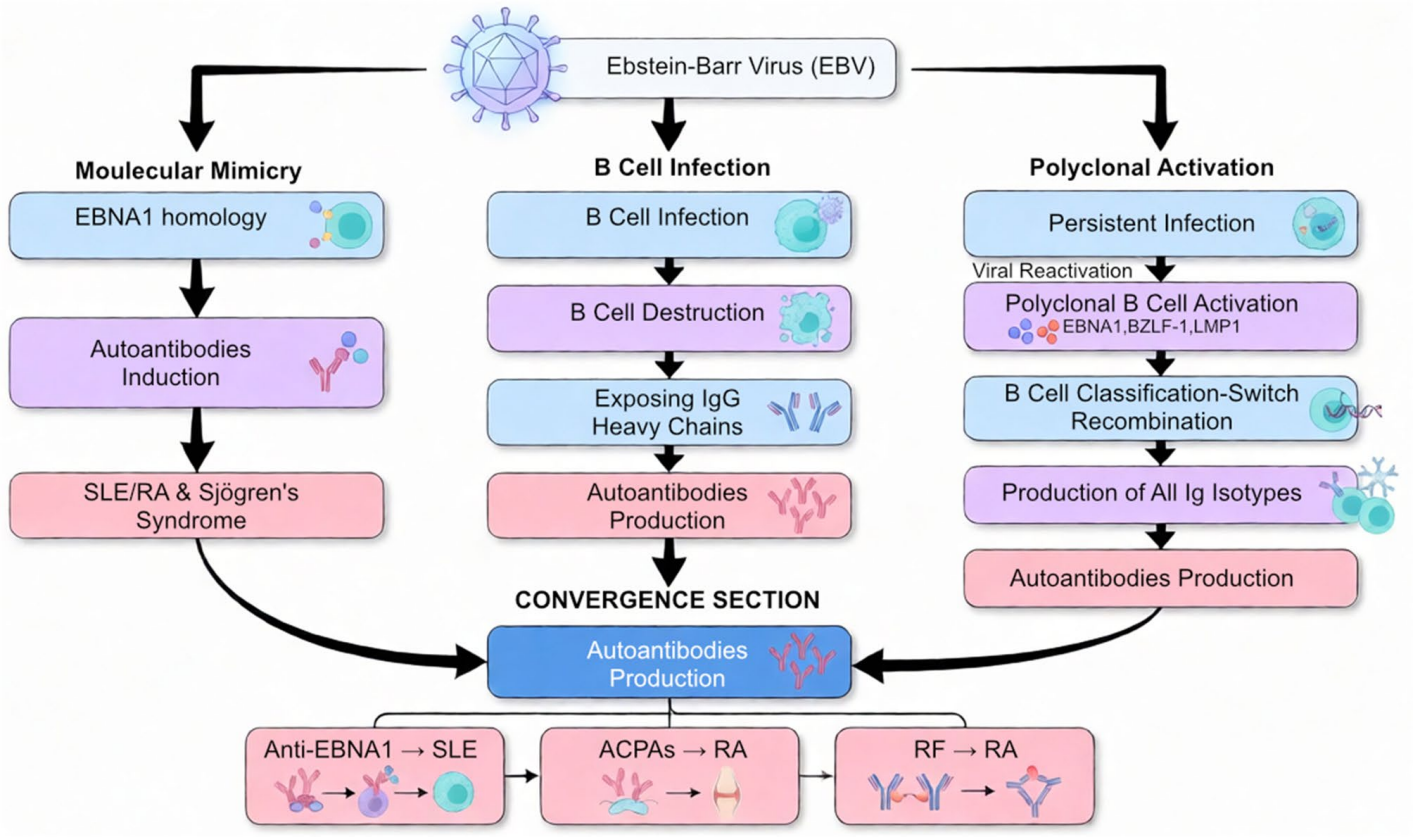
EBV infection mechanisms contributing to autoimmune diseases. SLE: systemic lupus erythematosus (SLE), RA: rheumatoid arthritis

Several other mechanisms have also been suggested to explain how EBV may contribute to RA, including bystander activation and persistent recurrent infection of synovial B cells. In addition, rheumatoid factors (RFs) have been found to target hidden epitopes linked to IgG heavy chains, which might become exposed following the destruction of EBV-infected B cells. Certain HLA-DRB1 alleles that carry the shared epitope (SE) motif have also been identified as strong binding partners for EBV gp42, facilitating the survival of EBV-infected B cells that express these MHC-II molecules [60]. Other autoimmune diseases, such as GBS and Sjogren’s Syndrome, have also been linked to EBV infection [61]. Sjogren’s Syndrome is a systemic autoimmune disorder characterized by the gradual destruction of salivary and lacrimal glands. Various environmental factors have been linked to its development, including vitamin D deficiency, smoking, exposure to silica dust, and viral infections—most notably infection with EBV [62]. Although the detailed mechanisms behind Sjogren’s Syndrome are less well studied, it is thought that they share similar underlying pathways. It is noteworthy that RA and Sjogren’s Syndrome often occur together. Importantly, Sjogren’s Syndrome primarily affects epithelial tissues such as the salivary and lacrimal glands, which are also the main sites for EBV infection. This overlap in tissue targets reinforces the proposed association between EBV infection and the development of Sjogren’s Syndrome [63] (Fig. 2).

Overall, EBV infects B lymphocytes and persists in them for a long time, leading to chronic activation of the immune system and the production of autoantibodies. As a result, tissue damage and chronic inflammation occur, creating a favorable environment for genetic mutations and the growth of abnormal cells. Therefore, the relationship between EBV, autoimmunity, and cancer develops through a cycle of inflammation and chronic immune dysregulation [64].

## Hepatitis B virus (HBV)

Hepatitis B virus (HBV) is a hepatotropic DNA virus in the Hepadnaviridae family. After infection, the viral genome is converted into covalently closed circular DNA (cccDNA), which persists in hepatocyte nuclei and provides a reservoir for long-term infection [65]. While HBV can remain dormant, reactivation occurs during immunosuppression and may influence extrahepatic immune processes [66]. Importantly, chronic HBV infection has been associated with autoimmune phenomena through multiple immunopathogenic mechanisms.

### HBV and autoimmune hepatitis (AIH)

Although the etiology of AIH remains unclear, viral infections, including HBV, have been proposed as possible triggers. Several mechanisms support a role for HBV in AIH development. First, molecular mimicry has been suggested, as structural similarities between HBV antigens (such as HBcAg or HBsAg) and host liver proteins can induce cross-reactive T-cell and B-cell responses [67, 68]. Second, HBV antigens expressed on hepatocyte surfaces may act as persistent immune targets, driving autoreactive cytotoxic T lymphocyte activity. Chronic HBV infection is also associated with reduced regulatory T-cell activity, impaired suppressor T-cell function, and increased pro-inflammatory cytokine release, all of which favor loss of tolerance and autoantibody production [69, 70]. Furthermore, HBV infection can induce aberrant expression of MHC molecules on hepatocytes, thereby enhancing the presentation of self-peptides to autoreactive T cells. Patients with concurrent AIH and HBV infection generally have better outcomes compared to those co-infected with HCV [69]. Collectively, these findings provide a mechanistic basis for viral-induced autoimmunity in the liver.

### HBV and Guillain-Barré syndrome (GBS)

Autoimmune neurological complications have also been reported in association with HBV infection. Approximately 1% of Guillain-Barré syndrome (GBS) cases have been reported in association with acute hepatitis caused by various hepatitis viruses (A, B, C, D, and E), and occasionally even with chronic hepatitis B (CHB) infection [71]. However, the precise mechanism by which chronic HBV infection leads to peripheral nerve damage in GBS remains unclear.

Neuropathological studies indicate that circulating HBsAg-immune complexes may cross the blood–nerve barrier, deposit in peripheral nerves, and initiate complement activation and local inflammation, ultimately leading to demyelination and axonal injury [72]. The temporal correlation between elevated immune complex levels and neurological symptom onset supports this immune complex-mediated mechanism. In addition, bystander activation and polyclonal T-cell responses during HBV infection may further contribute to the breakdown of peripheral tolerance.

**Fig. 3:**
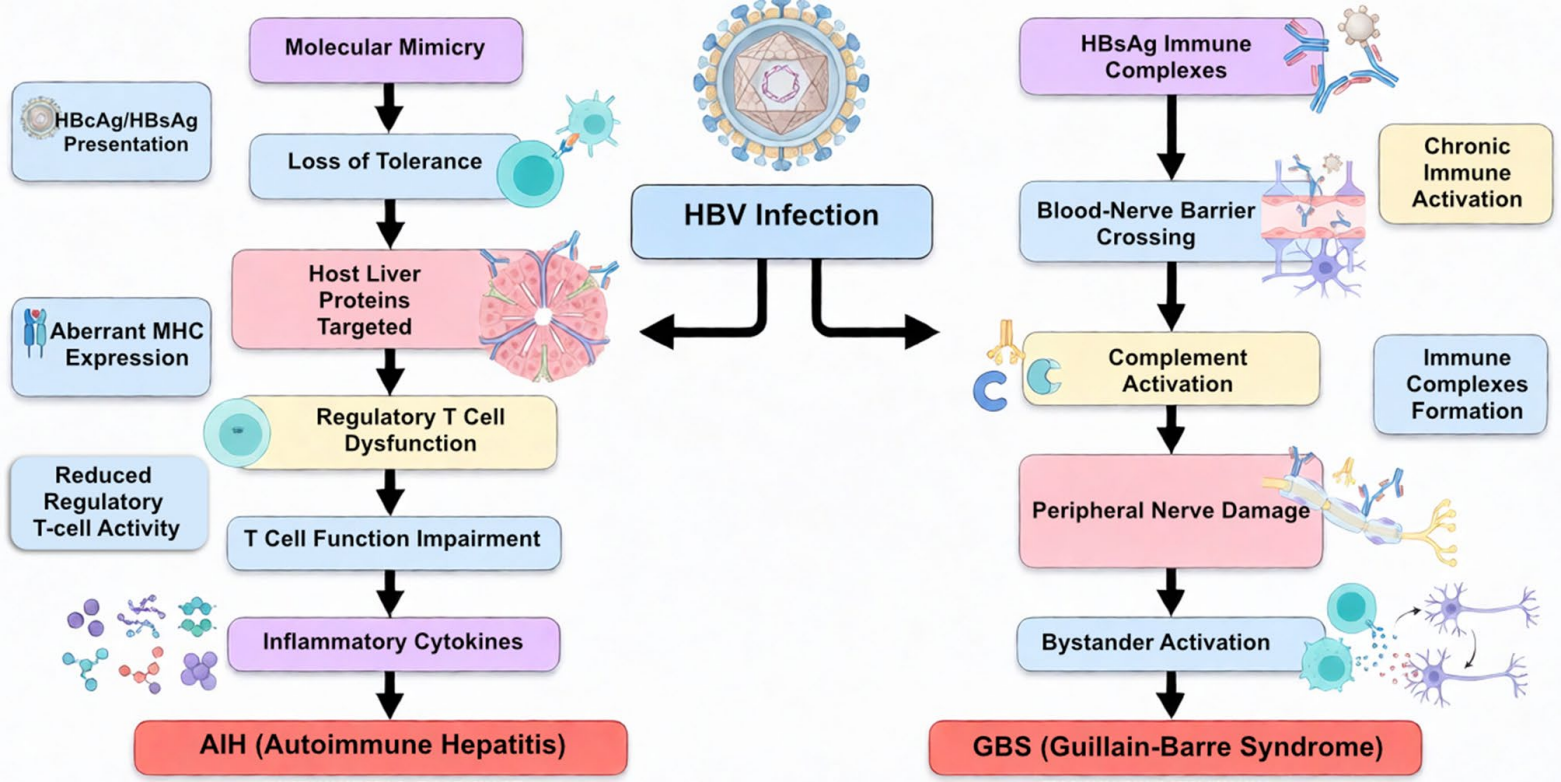
HBV infection mechanisms linked to Autoimmune Hepatitis (AIH) and Guillain-Barré Syndrome (GBS)

Taken together, evidence suggests that HBV can promote autoimmunity by triggering molecular mimicry, immune complex deposition, and impaired immune regulation, linking chronic viral persistence to the onset or exacerbation of autoimmune hepatitis and Guillain-Barré syndrome (Fig. 3).

Overall, the hepatitis B virus (HBV), by causing chronic liver infection, can lead to continuous immune stimulation and the development of autoimmune responses. During prolonged infection, immune cells mistakenly attack the body’s own liver cells, resulting in chronic inflammation, tissue damage, and fibrosis. Persistent inflammation and repeated cellular regeneration create a favorable environment for the accumulation of genetic mutations, ultimately increasing the risk of hepatocellular carcinoma. Therefore, HBV plays a crucial role in linking autoimmunity and cancer through the induction of autoimmune responses and sustained inflammation [73].

## Human herpesvirus 8 (HHV8)

Human Herpesvirus 8 (HHV-8), also known as Kaposi’s sarcoma-associated herpesvirus (KSHV), is a member of the herpesvirus family, and its association with autoimmune diseases is an area of ongoing research. While HHV-8 is primarily known for its role in Kaposi’s sarcoma, it has also been implicated in the pathogenesis of certain autoimmune diseases, particularly in immunocompromised individuals [74]. The potential biological mechanisms linking HHV-8 to the onset and progression of autoimmune.

Diseases consist of Immune Dysregulation Induced by HHV-8 that include latent infection, altered immune function, and cytokine imbalance. During latent Infection, HHV-8 can establish latency in a variety of immune cells, including B cells and endothelial cells. During latency, the virus can express certain viral proteins, such as LANA (Latency-associated nuclear antigen) and vFLIP (viral FLICE inhibitory protein), which modulate host immune responses [75].

HHV-8 infection can suppress host immune responses by interfering with antigen presentation, disrupting T-cell activation, and inhibiting apoptosis of infected cells. This immune suppression can impair the body’s ability to regulate self-reactivity, potentially contributing to the development of autoimmune diseases.

Infection with this virus, can lead to the production of pro-inflammatory cytokines such as IL-6 and TNF-α. Chronic inflammation induced by these cytokines can drive autoimmune responses, where the immune system attacks its own tissues. The presence of these cytokines may also promote the expansion of autoreactive B and T cells [76]. In addition, molecular Mimicry and Cross-Reactivity of HHV-8 can induce immune responses against its own proteins, leading to the production of autoantibodies that cross-react with host tissues. This phenomenon, known as molecular mimicry, occurs when the viral antigens share structural similarities with host self-antigens, causing the immune system to attack its own cells. Studies suggest that HHV-8 infection may trigger autoimmune responses through molecular mimicry, where the viral proteins resemble human proteins, particularly in autoimmune conditions such as SLE and rheumatoid arthritis (RA) [77].

Moreover, immune Complex Formation due to the presence of viral antigens in tissues can lead to the formation of immune complexes—combinations of viral antigens and antibodies. These complexes can accumulate in various tissues, activating the complement system and causing tissue damage through inflammation. This contributes to the progression of autoimmune diseases, such as lupus nephritis and rheumatoid arthritis [78].

In addition, endothelial Cell Dysfunction and Vasculitis HHV-8 has a strong affinity for endothelial cells and is associated with the development of vascular complications. Endothelial cells play a crucial role in regulating immune responses and maintaining vascular homeostasis. HHV-8 infection in these cells can lead to their dysfunction, promoting vascular inflammation (vasculitis) and potentially contributing to autoimmune conditions such as systemic vasculitis. The virus can induce the expression of adhesion molecules on endothelial cells, promoting the recruitment of inflammatory immune cells, leading to further tissue damage and the exacerbation of autoimmune processes [79].

B Cell Activation and Autoantibody Production of HHV-8 has been shown to directly infect B cells, promoting their activation and proliferation. This leads to an increase in the production of autoantibodies against self-antigens. These autoantibodies are a hallmark of many autoimmune diseases, including lupus and Sjögren’s syndrome. The virus may also contribute to B cell dysregulation, enhancing the formation of germinal centers and the development of autoimmune B cells that produce pathogenic antibodies. Additionally, viral proteins such as vIL-6 (viral interleukin-6) can mimic host IL-6, further contributing to B cell activation and the autoimmune response [80].

Increased Risk in Immunocompromised Hosts Immunocompromised individuals, such as those with HIV/AIDS, are at greater risk for HHV-8-associated diseases. In these individuals, HHV-8 can trigger chronic immune activation and persistent inflammation, which can lead to the development of autoimmune diseases. The virus may exploit the weakened immune system, leading to a breakdown of self-tolerance and the emergence of autoimmune responses. Additionally, the chronic viral infection can result in immune exhaustion, where immune cells become less responsive to infections and fail to regulate autoimmune reactions effectively [81]. Also, alteration of T Cell Responses of HHV-8 can modulate T cell responses by expressing viral proteins that interfere with the normal functioning of T helper cells (Th1 and Th17), which are involved in the immune response to infections and in autoimmunity. Dysregulation of these T cell subsets can promote autoimmune responses, especially in diseases such as multiple sclerosis or rheumatoid arthritis, where Th17 cells are often implicated [82]. The virus’s ability to infect multiple immune cell types and modulate host immune responses makes it a potential contributor to the development and progression of autoimmune diseases.

Castleman disease (CD) is an uncommon, nonclonal lymphoproliferative disorder that is diagnosed based on characteristic histopathological findings [83]. In a subset of patients with multicentric Castleman disease (MCD), there is evidence of polyclonal proliferation of plasmablasts infected with human herpesvirus 8 (HHV8), a condition referred to as “HHV8-associated MCD.” This form is primarily observed in individuals with compromised immune systems. The affected plasmablasts generally express immunoglobulin M (IgM) along with lambda light chains and are typically located within the mantle zone of lymph nodes, where they are accompanied by a dense infiltrate of polyclonal plasma cells [84].

**Fig. 4:**
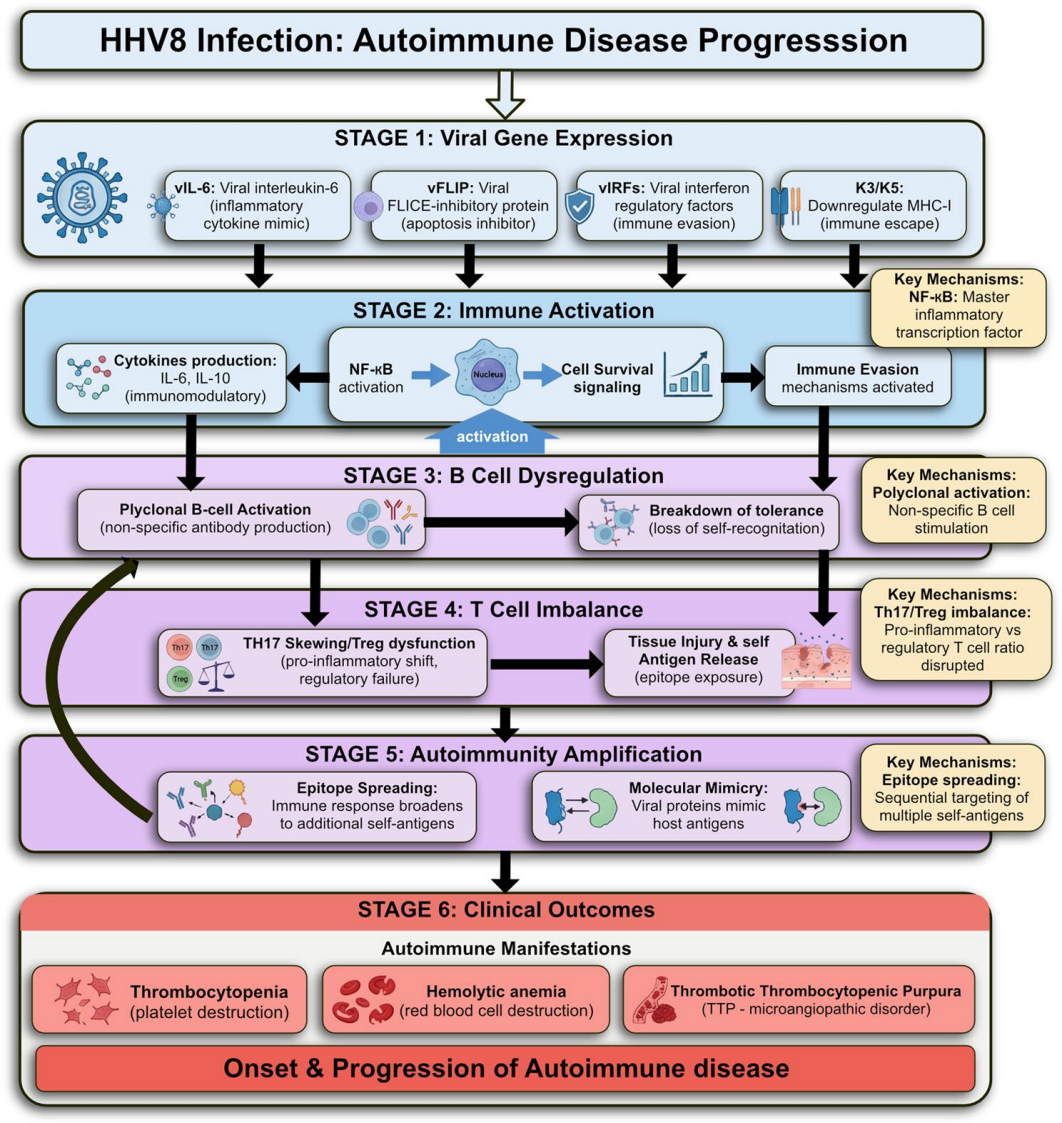
HHV-8 infection mechanisms contributing to multicentric Castleman disease (MCD) and autoimmune manifestations. IL-6: interleukin-6, TTP: thrombotic thrombocytopenic purpura

This disorder can become life-threatening due to complications such as hemophagocytic lymphohistiocytosis and other syndromes associated with excessive cytokine release [85]. During disease flares, various autoantibodies may appear, and autoimmune manifestations such as thrombocytopenia, hemolytic anemia, and thrombotic thrombocytopenic purpura have been documented in affected individuals [86] (Fig. 4).

This virus infects endothelial cells and certain immune cells, leading to the activation of inflammatory pathways and the release of autostimulatory cytokines, which result in chronic inflammation and autoimmune responses. Persistent inflammation and prolonged cellular stimulation create favorable conditions for uncontrolled proliferation and genetic mutations. Consequently, this process can lead to the formation of vascular tumors such as Kaposi’s sarcoma [87].

## Merkel cell polyomavirus

Merkel cell carcinoma (MCC) is a rare but highly aggressive neuroendocrine tumor. Iatrogenic immunosuppression has been recognized as a contributing risk factor in the development of this malignancy. The mechanistic link is not as well-studied, but several plausible biological pathways are proposed. MCPyV large T (LT) and small T (sT) antigens share sequence or structural homology with host proteins. Virus-specific immune responses can cross-react with self-antigens, activating autoreactive T and B cells. This event cause Producing of autoantibodies or cytotoxic responses against self-tissues [88]. Also, MCPyV establishes persistent infection in skin and possibly other tissues. Long-term presence of viral antigens sustains chronic activation of innate and adaptive immune pathways. So, bystander activation of autoreactive lymphocytes and chronic tissue inflammation observed [89]. In addition, MCPyV infection modifies MHC class I and class II expression in infected cells that leads to aberrant presentation of self-peptides alongside viral peptides, and Breakdown of immune tolerance and enhanced recognition of self-antigens [90]. Moreover, MCPyV may interact with B cells through viral capsid proteins, and Causes polyclonal B-cell activation and autoantibody generation (e.g., ANA, anti-thyroid antibodies) [91]. Approximately 10% of MCC cases occur in immunosuppressed individuals, including those with underlying hematologic disorders, HIV/AIDS, or who have undergone organ transplantation [92].

The advent of biologic disease-modifying antirheumatic drugs (bDMARDs) has significantly transformed the treatment landscape for various rheumatic conditions, such as rheumatoid arthritis (RA) and spondyloarthritis. Robust evidence from randomized clinical trials and large-scale observational studies has confirmed the effectiveness of bDMARDs in achieving substantial clinical improvement, frequently leading to disease remission. These therapies not only reduce symptoms but also play a crucial role in preventing long-term functional impairment and slowing or even halting the progression of joint damage seen on radiographic imaging [93].

**Fig. 5:**
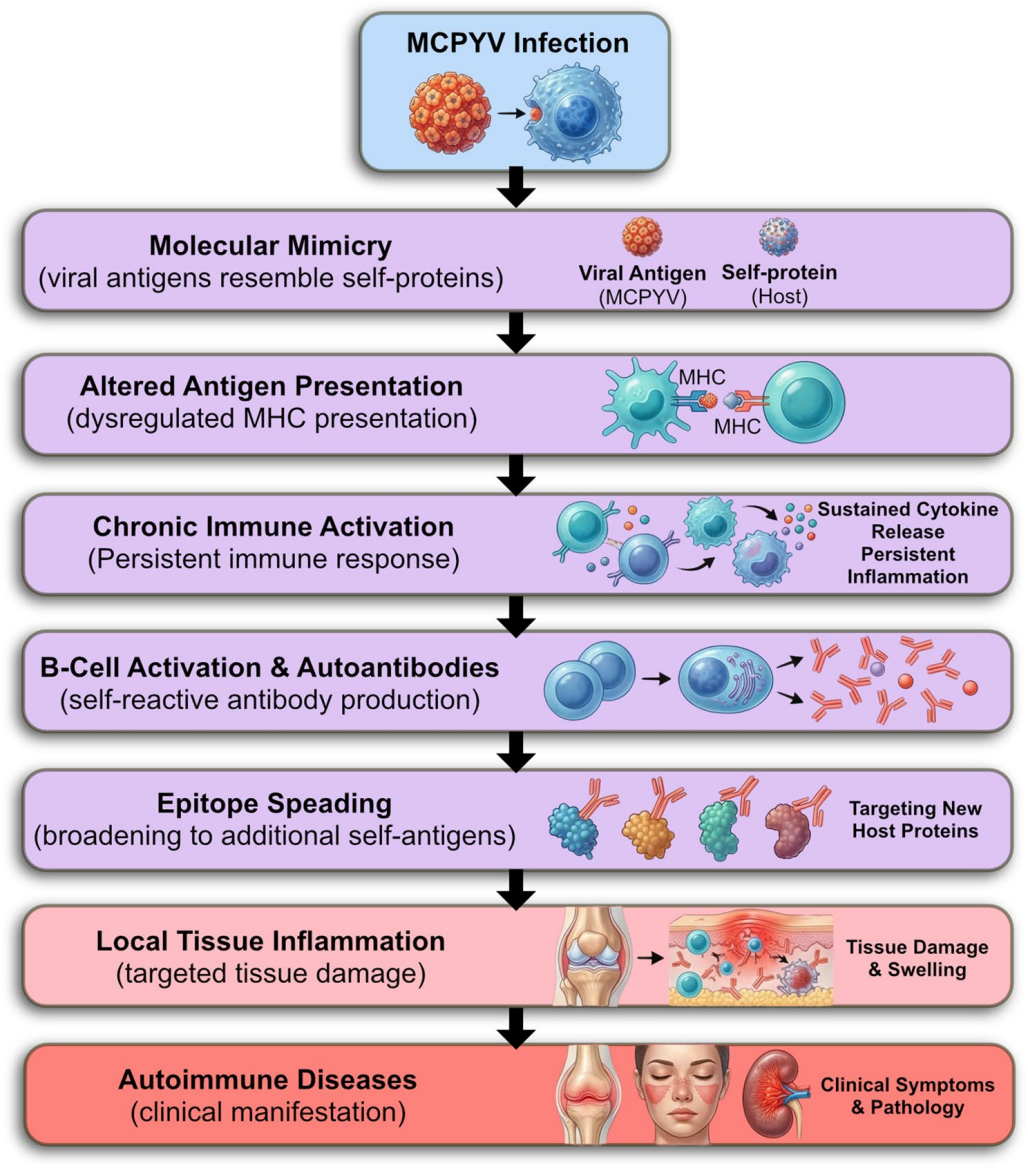
MCPyV infection mechanisms contributing to Merkel cell carcinoma (MCC) and autoimmune diseases

Numerous comprehensive reviews have evaluated and compared the adverse effects associated with different bDMARDs—including TNF inhibitors, IL-1 and IL-6 antagonists, as well as monoclonal antibodies targeting CD28 and B cells—across a range of rheumatic diseases [94, 95] (Fig. 5).

Merkel cell polyomavirus (MCPyV) usually exists in the body in a latent state, but under conditions of immune dysfunction or suppression, it can become active and trigger autoimmune and oncogenic consequences. By affecting immune cells and their regulatory pathways, this virus induces chronic inflammatory responses and sometimes the production of autoantibodies, leading to tissue damage and immune instability. Ultimately, this process can lead to the development of Merkel cell carcinoma a rare but highly aggressive skin tumor [96].

## Hepatitis C virus (HCV)

The relationship between chronic hepatitis C virus (HCV) infection and autoimmune conditions is complex, with HCV potentially acting as a trigger for autoimmune phenomena or diseases in certain individuals.

### HCV and autoimmune hepatitis (AIH)

While there is evidence supporting the successful treatment of HCV using direct-acting antivirals (DAAs) in patients co-infected with autoimmune hepatitis (AIH), there are also isolated reports of AIH emerging during or following DAA therapy [97]. In chronic HCV patients, smooth muscle antibodies (SMA) and anti-nuclear antibodies (ANA) are often detectable in the serum. However, their presence does not appear to significantly influence biochemical or histological findings [98]. These autoantibodies are also characteristic of type I AIH, which can make diagnosis challenging when other potential causes of liver disease, such as chronic HCV, are present [99]. HCV was first identified in 1987 as the causative agent of what was previously known as non-A, non-B hepatitis [100].

The mechanisms underlying this link are multifactorial and consist of immune dysregulation, chronic immune activation, and persistent viral infection. Some mechanistic pathway includes Molecular mimicry: HCV proteins specially NS3, NS5, and E2 glycoproteins share structural similarities with host proteins. Due to this similarity, T cells and antibodies generated against viral epitopes can cross-react with self-antigens (e.g., thyroid peroxidase, nuclear proteins), driving autoimmunity [101].

B-cell hyperactivation and autoantibody production is another way that HCV directly infects B cells or stimulates them via CD81 and other coreceptors, that leads to polyclonal and oligoclonal B-cell expansion, with production of autoantibodies (e.g., rheumatoid factor, anti-thyroid antibodies, ANA). This underlies HCV-associated cryoglobulinemia and contributes to systemic autoimmune diseases [102]. Persistent HCV infection maintains chronic IFN-α, TNF-α, and IL-6–mediated immune activation, even in non-hepatic tissues that cause the non-specific activation of autoreactive T cells (bystander activation), breakdown of tolerance, and tissue inflammation [103]. In addition, defective regulatory T cells (Tregs) due to the HCV infection disrupts Treg numbers and function, and impaired control of autoreactive effector T cells, leading to loss of tolerance and enhanced autoimmunity [104].

Chronic inflammation and hepatocyte damage release intracellular antigens (nuclear, mitochondrial, cytoskeletal). Antigen-presenting cells present these self-antigens to T cells, broadening the autoimmune response beyond the initial viral target [105].

Direct tissue-specific effects is another event in HCV infection that can infect thyroid tissue, promoting local inflammation and autoimmune thyroiditis [106]. Also, HCV triggers lymphocytic infiltration resembling Sjögren’s syndrome. In addition, immune complex deposition of HCV antibody complement contributes to vasculitic manifestations [107]. Generally, HCV infection sustains chronic immune stimulation, causes molecular mimicry, and drives B-cell dysregulation. This environment fosters loss of tolerance and development of autoantibodies, which combined with local viral tropism led to a spectrum of autoimmune diseases.

### HCV and autoimmune thyroid disorders

During chronic HCV infection, symptoms of both hypothyroidism and hyperthyroidism may arise, with Hashimoto’s thyroiditis being the most commonly reported thyroid disorder. Treatment with interferon-alpha (IFNA) is a significant risk factor for the development of thyroid dysfunction. IFNA may induce autoimmunity through several molecular mechanisms, including the presence of polymorphisms in the feed-forward loop of IFNA production, alterations in IFNA-signaling pathways, and a mutually reinforcing feedback loop involving IFNA and estrogen receptor-α. Elevated levels of IFNA exert multiple immunomodulatory effects, including the activation of both the innate and adaptive immune responses [108].

### HCV-associated arthropathy vs. rheumatoid arthritis (RA)

Polyarthritis can also occur in the context of HCV infection and is sometimes linked to mixed cryoglobulinemia [109, 110]. One study investigated the diagnostic value of anti-mutated citrullinated vimentin (anti-MCV) antibodies—proposed for inclusion in RA diagnostic criteria—by measuring anti-MCV, anti-cyclic citrullinated peptide (anti-CCP) antibodies, rheumatoid factor, and cryoglobulins. The most common clinical presentation was symmetrical polyarthralgia, with the shoulders, knees, wrists, and metacarpophalangeal joints being most frequently affected. In HCV-related arthropathy, anti-MCV antibodies were positive in 30% of cases, anti-CCP antibodies in 0%, and rheumatoid factor in 73.3%. In contrast, in RA patients, positivity rates were 93.3% for anti-MCV, 96.7% for anti-CCP, and 86.7% for rheumatoid factor. These findings highlight the diagnostic value of anti-CCP antibodies in distinguishing between HCV-associated arthropathy and RA [111].

### HCV and Sjogren’s Syndrome (SS)

**Fig. 6:**
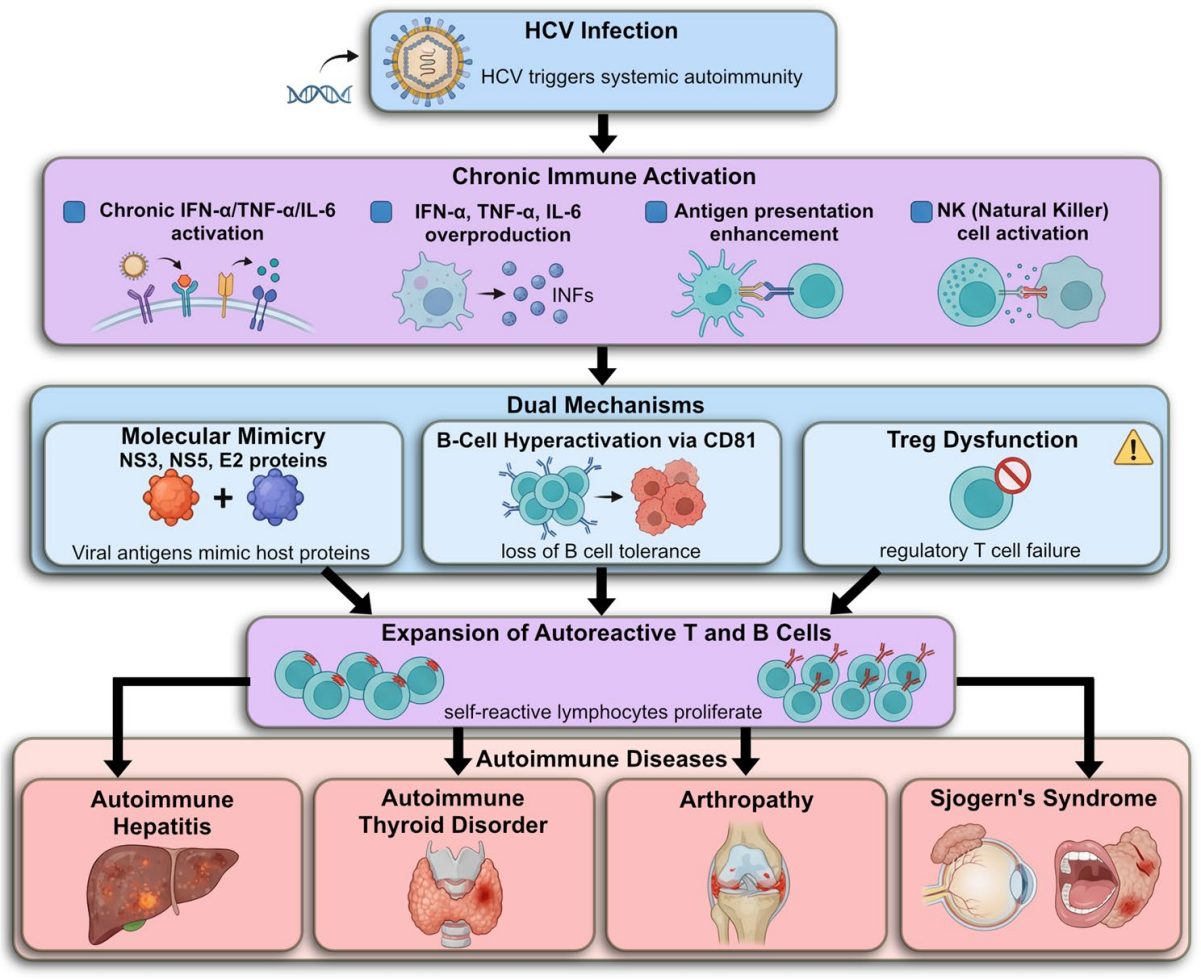
HCV-induced immune dysregulation contributing to autoimmune diseases and lymphoproliferative disorders

Sjogren’s syndrome often presents with non-erosive arthritis and systemic symptoms such as fatigue. Laboratory results typically reveal positive SSA and/or SSB autoantibodies, often in conjunction with rheumatoid factor. SS may occur independently or secondary to other autoimmune diseases like RA or systemic lupus erythematosus [112]. Although a direct causal link between HCV and SS has not been firmly established, many studies have explored the possibility of an association. HCV-related SS tends to show distinctive immunologic features, including high prevalence of anti-mitochondrial antibodies, anti-gastric parietal cell antibodies, hypocomplementemia, and cryoglobulinemia, along with a lower prevalence of SSA autoantibodies [113]. Despite these differences, some theories propose shared mechanisms between HCV-associated SS and lymphoproliferative disorders. Further research is needed to clarify the precise role of HCV infection in the development of Sjogren’s syndrome [114] (Fig. 6).

The hepatitis C virus (HCV), by causing chronic infection and continuous immune stimulation, plays a significant role in the development of autoimmune diseases and cancer. By infecting liver cells and engaging immune cells, this virus leads to excessive cytokine secretion and the production of autoantibodies, resulting in chronic inflammation and gradual liver tissue damage, ultimately leading to liver cancer (hepatocellular carcinoma) [115].

## Human T-cell Lymphotropic Virus Type 1 (HTLV-1)

Infection with HTLV-1 (Human T-cell Lymphotropic Virus Type 1) primarily targets lymphocytes, which circulate throughout the body, affecting various organs and leading to diverse clinical manifestations—particularly among underserved populations lacking adequate access to healthcare.

There is also a growing of evidence suggesting a link between HTLV-1 infection and the development or progression of autoimmune diseases. However, the precise mechanisms of pathogenesis remain incompletely understood. The viral proteins HBZ (HTLV-1 basic leucine zipper factor) and Tax play critical roles in the persistence of infection and contribute to pathogenesis by promoting cell proliferation and modulating inflammatory and immune responses associated with the virus’s clinical outcomes [116].

The HTLV-1 viral proteins, such as Tax and HBZ, may mimic host antigens. The immune system may misidentify self-antigens as foreign due to molecular mimicry, leading to autoimmune responses [117].

HTLV-1-infected T-cells express Tax protein, which promotes the survival and proliferation of these cells. Tax can also dysregulate immune signaling pathways, particularly those involving NF-kB and AP-1, leading to enhanced T-cell survival and abnormal immune responses [118].

HTLV-1 is classified as a type C retrovirus, belonging to the Deltaretrovirus genus, the Retroviridae family, and the Orthoretrovirinae subfamily [119]. It has been associated with several diseases, including HAM/TSP (HTLV-1-associated myelopathy/tropical spastic paraparesis) and autoimmune disorders such as Sjögren’s syndrome (SS), uveitis, and arthropathies. These conditions are often linked to disruptions in immune regulation [120]. HTLV-1 primarily infects CD4 + T-lymphocytes and is capable of modulating their function.

Infected CD4 + T-cells, that lead to immune dysregulation and excessive production of pro-inflammatory cytokines, such as IL-2, IFN-γ, and TNF-α [121].

Given that CD4 + T cells play a vital role in regulating adaptive immune responses, changes in their behavior may provoke inflammatory reactions that compromise immune tolerance and contribute to the development of autoimmunity [122].

Several studies have demonstrated that the HTLV-1 Tax protein affects multiple transcription factors, including CREB/ATF, NF-κB, AP-1, SRF, and NFAT (nuclear factor of activated T-cells). Tax also interferes with signaling pathways involving PDZ domain-containing proteins, such as Rho-GTPases, and the Janus kinase (JAK)/signal transducer and activator of transcription (STAT) pathway.

These disruptions impact TGF-β (transforming growth factor β) signaling, influencing cell proliferation and activation, as well as the expression of cytokines and viral proteins [123, 124]. Moreover, FOXP3 (forkhead box P3)—a transcription factor essential for the function, differentiation, and homeostasis of regulatory T cells (Tregs)—has been found to be dysregulated in individuals infected with HTLV-1. This dysregulation may lead to loss of immune tolerance and contribute to the development of autoimmune conditions [125, 126].

In a study conducted by Yamano et al., it was shown that persistent immune activation driven by Tax in HAM/TSP patients may result in a decrease in the population of CD4 + CD25+FOXP3 + T cells, which normally exert suppressive functions. In contrast, there was an accumulation of CD4 + CD25+FOXP3 − T cells, potentially exacerbating the pathogenic process of HAM/TSP. The authors further demonstrated that the frequency of CD4 + CD25+FOXP3 − T cells was significantly elevated in these patients and was associated with the clinical severity of HAM/TSP [127].

The virus has developed strategies to evade the immune system, including downregulation of major histocompatibility complex (MHC) molecules and inhibition of apoptosis in infected T-cells [128].

By evading immune clearance, HTLV-1 promotes persistent infection that could alter immune responses and increase the risk of autoimmune disease development [129].

Overall, viral protein-mediated changes in signaling pathways and transcription factor activation play a critical role in disturbing immune homeostasis, generating a cytokine environment that alters immune cell phenotypes.

### HTLV-1 and rheumatoid arthritis (RA)

In RA, disease progression and severity are largely influenced by the migration of T lymphocytes into the synovial tissue. Earlier studies have reported synovial proliferation and T cell infiltration in HTLV-1-infected individuals who subsequently develop RA. Additionally, the presence of HTLV-1 proviral DNA has been demonstrated in synovial fluid and tissues, with infection of T cells in both the synovium and synovial lining [130].

Nishioka et al. reported the presence of Tax mRNA in synovial cells from patients with HTLV-1-associated arthropathy. Tax protein appears to stimulate cell proliferation and induce the production of pro-inflammatory cytokines [131]. Similarly, to what is observed in HTLV-1-associated HAM/TSP, lymphocyte migration into the central nervous system (CNS) has been reported in these patients. This migration may be related to the patient’s viral load. Yakova et al. observed that HTLV-1-infected individuals with connective tissue diseases or RA had higher viral loads compared to asymptomatic carriers. Interestingly, the viral load in these patients was similar to that of individuals with HAM/TSP. Moreover, viral load levels in the synovium were higher in RA patients than in asymptomatic individuals [132, 133].

### HTLV-1 and Sjogren’s Syndrome (SS)

Sjogren’s Syndrome is a systemic autoimmune disorder primarily characterized by xerostomia (dry mouth) and xerophthalmia (dry eyes) due to lymphocytic infiltration of salivary and lacrimal glands, resulting in ductal destruction. Patients with SS typically exhibit antinuclear antibodies (ANA) and other autoantibodies, including anti-SS-A (Ro) and anti-SS-B (La). The development of the disease is influenced by genetic and hormonal factors [134]. Moreover, several viral infections, including HTLV-1, have been implicated in SS pathogenesis, with multiple studies reporting a higher prevalence of HTLV-1 among SS patients [135].

Nakamura et al. identified a high frequency of anti-HTLV-1 IgA antibodies in the salivary glands of patients with SS [136]. Additionally, SS patients infected with HTLV-1 showed more extensive mononuclear cell infiltration in affected tissues compared to uninfected individuals.

### HTLV-1 and systemic lupus erythematosus (SLE)

The relationship between HTLV-1 and SLE remains controversial [137, 138]. One proposed mechanism involves molecular mimicry through the endogenous retroviral sequence HRES-1, which may contribute to the development of SLE by promoting autoantibody production and the formation of immune complexes that deposit in tissues and trigger inflammation and complement activation—hallmarks of SLE [139].

Moreover, several studies have reported the expression of HTLV-1-related antigens in peripheral blood mononuclear cells of SLE patients following in vitro culture for three or more days. This observation suggests ongoing viral replication in these individuals, which may explain the high rates of HTLV-1 and HTLV-2 seropositivity seen in SLE patients [140].

**Fig. 7:**
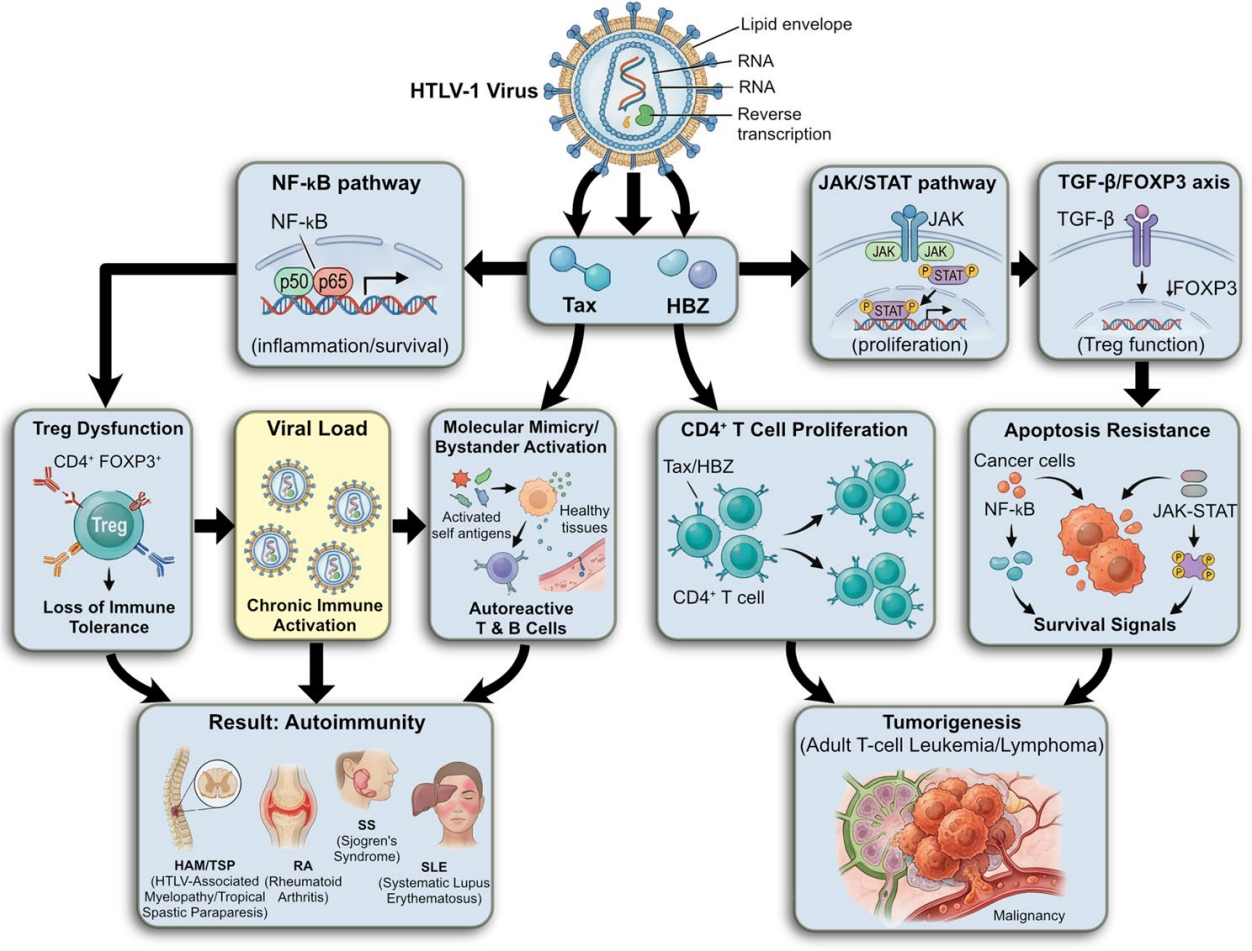
HTLV-1 infection mechanisms contributing to oncogenesis and autoimmune diseases. NF-κB: nuclear factor kappa B, JAK/STAT: Janus kinase/signal transducer and activator of transcription, TGF-β: transforming growth factor beta, Treg: regulatory T cell, FOXP3: forkhead box P3, HAM/TSP: HTLV-1–associated myelopathy/tropical spastic paraparesis, RA: rheumatoid arthritis, SS: Sjögren’s syndrome, SLE: systemic lupus erythematosus

Furthermore, elevated HTLV-1 proviral loads have been observed in patients with connective tissue disorders, and HTLV-1 DNA has been detected in both synovial fluid and CNS tissues of affected individuals [141] (Fig. 7).

Overall, HTLV-1 can facilitate the development of autoimmune diseases by infecting T cells and disrupting immune system function. This virus activates inflammatory pathways and alters the regulation of genes related to cell proliferation and survival, leading to abnormal immune responses and the production of autoantibodies, which in some individuals can lead to cancers such as Adult T-cell Leukemia/Lymphoma [142].

## Association of autoimmune disease and cancer

Autoimmune diseases arise when the immune system mistakenly targets the body’s own tissues, whereas cancer develops when the immune system fails to detect and eliminate abnormal or malignant cells [12]. The connection between altered immune responses in cancer progression and the presence of autoimmune diseases has drawn increasing attention. The intricate relationship between chronic inflammation, immune system dysregulation, and the development of cancer has led to a growing understanding of the links between autoimmune diseases and malignancies.

Interestingly, early-stage cancers and autoimmune diseases often share inflammatory features, and these overlapping patterns are also observed in advanced cancers. This similarity may explain the increased incidence of various cancers in patients with pre-existing autoimmune conditions. Despite this, some data suggest that such patients may experience lower cancer-specific mortality rates [143].

Patients with autoimmune diseases (AIDs) appear to be more prone to developing different types of cancers. For example, earlier research has reported a higher occurrence of malignancies in individuals with rheumatoid arthritis (RA). The underlying mechanisms of RA involve abnormalities in the innate immune system, including cytokine imbalances and cellular dysfunctions, which may contribute to cancer development and progression.

In the case of Sjögren’s syndrome (SS), there is a documented increase in the risk of several malignancies—particularly non-Hodgkin lymphoma (NHL), where in some studies the risk has been reported to be more than ten times higher than in the general population. Similarly, systemic lupus erythematosus (SLE) has been linked to a variety of cancers, including both organ-specific tumors and hematologic malignancies.

Scleroderma (Sc) is another autoimmune disorder in which cancer risk is elevated. Notably, patients with scleroderma have shown a higher prevalence of certain cancers, especially breast and lung cancer—an association that has been consistently observed and well-documented in numerous studies [144].

### Increased cancer risk in autoimmune disease patients

Individuals diagnosed with autoimmune diseases face an increased risk of developing various types of cancer. This elevated risk is thought to stem from chronic inflammation, dysregulation of the immune system, and long-term use of immunosuppressive medications.

- **Rheumatoid Arthritis (RA)**

Early research identified a higher prevalence of cancer among patients with RA. The underlying mechanisms of the disease—such as ongoing inflammation, abnormal cytokine activity, and sustained immune cell activation—are believed to play a role in promoting tumor formation [145].

- **Sjögren’s Syndrome (SS)**

Patients with Sjögren’s syndrome are particularly vulnerable to hematologic malignancies, especially non-Hodgkin lymphoma (NHL). In some reports, the risk of developing NHL is up to tenfold higher than that of the general population. Chronic B-cell stimulation and abnormal lymphocyte proliferation seen in SS are major factors contributing to this increased susceptibility [146].

- **Systemic Lupus Erythematosus (SLE)**

SLE is associated with a higher incidence of both solid tumors and blood-related cancers. Chronic immune activation, impaired clearance of apoptotic cells, and the long-term administration of immunosuppressants are believed to collectively contribute to this elevated cancer risk.

- **Systemic Sclerosis (Scleroderma**,** Sc)**

In patients with systemic sclerosis, the risk of developing malignancies—particularly breast and lung cancers—is significantly increased. Persistent tissue injury, progressive fibrosis, and abnormal immune responses within affected organs are thought to foster conditions conducive to cancer development [147].

## Conclusion

Viral infections play a crucial role in triggering autoimmune processes, with oncogenic viruses contributing through complex and multifaceted mechanisms. Current evidence indicates that viral oncoproteins activate key pathways involved in autoimmune responses, especially in diseases that share molecular connections with cancer. Inflammation acts as a common driving force in both autoimmune diseases and early-stage cancer, while paradoxically inhibiting the progression of advanced cancer. Consequently, autoimmune diseases may promote cancer initiation and early tumor development, increasing the overall risk of malignancy. However, the strength of the association between autoimmune diseases and cancer varies depending on the specific autoimmune disorder, cancer type, and population studied. A deeper understanding of these interconnected pathways could provide valuable insights for improved disease management and therapeutic strategies.

## Data Availability

Data sharing not applicable to this article as no datasets were generated or analysed during the current study.
